# *P* value and Bayesian analysis in randomized-controlled trials in child health research published over 10 years, 2007 to 2017: a methodological review protocol

**DOI:** 10.1186/s13643-021-01622-8

**Published:** 2021-03-10

**Authors:** Alex Aregbesola, Allison Gates, Amanda Coyle, Shannon Sim, Ben Vandermeer, Megan Skakum, Despina Contopoulos-Ioannidis, Anna Heath, Lisa Hartling, Terry P. Klassen

**Affiliations:** 1 The Children’s Hospital Research Institute of Manitoba, 513-715 McDermot Avenue, John Buhler Research Centre, Winnipeg, Manitoba R3E 3P4 Canada; 2 Department of Pediatrics and Child Health, Max Rady College of Medicine, Rady Faculty of Health Sciences, University of Manitoba, Winnipeg, MB Canada; 3 Department of Pediatrics and the Alberta Research Centre for Health Evidence (ARCHE), University of Alberta, Edmonton, Canada; 4 Department of Pediatrics, Division of Infectious Diseases, Stanford University School of Medicine and Meta Research Innovation Center at Stanford (METRICS), Stanford, CA USA; 5 The Hospital for Sick Children and the University of Toronto, Toronto, ON Canada; 6 University College London, London, UK

**Keywords:** *P*value, Frequentist, Bayesian, Analysis, Child health, Randomized-controlled trials

## Abstract

**Background:**

There is an unresolved debate about the reliability of the interpretation of *P* value. Some investigators have suggested that an alternative Bayesian method is preferred in conducting health research. As randomized-controlled trials (RCTs) are important in generating research evidence, we decided to investigate the extent, if any, the inferential statistical framework in published RCTs in child health research have changed over 10 years. We aim to examine the change in *P* value and Bayesian analysis in RCTs in child health research papers published from 2007 to 2017.

**Methods:**

We will search the Cochrane Central Register of Controlled Trials (Wiley) to identify relevant citations. We will leverage a pre-existing sample of child health RCTs published in 2007 (*n*=300) used in our previous study of reporting quality of pediatric RCTs. Using the same strategy and study selection methods, we will identify a comparable random sample of child health RCTs published in 2017 (*n*=300). Eligible studies will include RCTs in health research among individuals aged 21 years and below. One reviewer will select studies for inclusion and extract the data and another reviewer will verify these. Disagreements will be resolved by a discussion between reviewers or by involving another reviewer. We will perform a descriptive analysis of 2007 and 2017 samples and analyze the results using both the frequentist and Bayesian methods. We will present specific characteristics of the clinical trials from 2007 and 2017 in tabular and graphical forms. We will report the difference in the proportion of *P* value and Bayesian analysis between 2007 and 2017 to assess the 10-year change. Clustering around *P* values of significance, if observed, will be reported.

**Discussion:**

This review will present the difference in the proportion of trials that reported on *P* value and Bayesian analysis between 2007 and 2017 to assess the 10-year change. The implications for future clinical research will be discussed and this research work will be published in a peer-reviewed journal. This review has the potential to help inform the need for a change in the methodological approach from the null hypothesis significance test to Bayesian methods.

**Systematic review registration:**

Open Science Framework https://osf.io/aj2df

**Supplementary Information:**

The online version contains supplementary material available at 10.1186/s13643-021-01622-8.

## Background

Authors have continued to debate the reliance on *P* value alone in reporting and interpreting health research findings [1]. Chavalarias et al.’s [2] study from the USA examined the trend of *P* values and other statistical information reported in the entire MEDLINE database on biomedical research for over 25 years and found an increase in the reporting of *P* values over time. They also found that smaller *P* values were reported in the abstracts compared to the full-text, and the Bayesian methods were almost completely absent in the studies. Goodman et al.’s [3] report, also from the USA, which explored the properties and consequences of using Bayesian factors, found that the Bayes factor provides information about effect size and considers the alternative hypotheses of data compared to *P* value, which is computed with only the null hypothesis.

Accumulating studies have relentlessly highlighted the limitations and misconceptions of *P* values [4, 5]. One of such numerous misconceptions is the interpretation of a non-statistically significant difference (*P* value >5%) between two groups to mean that the null effect is most likely. This just means, however, that the null effect is statistically consistent with the observed results, including the range of effects in the confidence interval (CI) [4]. Likewise, equating statistical significance to clinical importance is erroneous because the difference may be too small to be clinically relevant. Sometimes, clinically relevant findings may not be statistically significant. While the use of *P* values may have a strong statistical history, compelling evidence showed that there is a need for complementary measures of evidence like effect sizes or replacing it with other inferential statistics such as Bayesian methods [6].

A study from Australia, which compared reporting research results with either the null hypothesis significance test (NHST, which is dependent on the *P* value) or confidence intervals (CIs), concluded that CIs elicit better interpretations if NHST is not invoked [7].

Some studies have also suggested that the subjective and arbitrary elements of *P* values are better clarified by Bayesian methods, which provide a more attractive alternative for better clinical trials [8]. A review that compared frequentist NHST with Bayesian statistics in health research concluded that NHST is susceptible to confident misinterpretation, while Bayesian methods provide direct answers to how confident we should be in our results [9]. In an attempt to limit or eradicate misinterpretations associated with frequentist statistics, some studies have called for a complete ban of *P* values and NHST [10].

Following unresolved debate about the reliability of *P* value interpretation and the increasing interest in Bayesian methods [8], we decided to investigate the extent, if any, the inferential statistical framework in child health research has changed over 10 years [11]. We aim to examine the change in *P* value and Bayesian analysis and clustering around *P* values of significance in randomized-controlled trials (RCTs) in child health research papers published from 2007 to 2017.

## Methods

The present protocol has been registered within the Open Science Framework (registration: https://osf.io/aj2df) and is being reported in accordance with the reporting guidance provided in the Preferred Reporting Items for Systematic Reviews and Meta-Analyses Protocols (PRISMA-P) statement [12] (see checklist in Additional file 1). Any amendments made to this protocol when conducting the study will be outlined in the Open Science Framework and reported in the final manuscript

### Search strategy and study selection

We will leverage a pre-existing sample of child health RCTs published in 2007 (*n* = 300) [11] used by our team in previous study of reporting quality of pediatric RCTs to answer our review question: What is the magnitude and direction of change in *P* value and Bayesian analysis reported in RCTs in child health research published over 10 years, if any? Details of the search strategy and study selection methods for the sample are available in our previous publications [11, 13]. We will replicate these methods to identify a comparable sample of child health RCTs published in 2017. The final sample will include 600 child-health RCTs, 300 published in each of 2007 and 2017.

To identify a sample of studies published in 2017, a research librarian will execute an updated literature search in the Cochrane Central Register of Controlled Trials (see Additional file 2). The Cochrane Central Register of Controlled Trials includes randomized and quasi-randomized controlled trials indexed in MEDLINE and EMBASE, hand-searched results, gray literature sources, and Cochrane Review Groups Specialized Registers of trials [14]. All retrieved records will be imported into EndNote (v. X9, Clarivate Analytics, Philadelphia, PA, USA) and exported to an Excel (v. 2016, Microsoft Corporation, Redmond, WA, USA) workbook for screening. We will randomly order the citations using the random numbers generator in Excel. Next, one reviewer will screen the titles and abstracts to identify the first 300 child health RCTs. These should be easily identifiable by title and abstract; however, in the unlikely (per experience) event that a record is deemed ineligible during data extraction, we will substitute it with the next relevant record. We will include the first 300 eligible citations from the randomly ordered list to make the sample size consistent with the previous publications [11, 12].

Eligible studies will include RCTs in health research conducted among individuals aged 21 years and below [15]. We will employ identical selection criteria used in the 2007 and 2012 samples to maintain consistency and comparability with earlier findings [11]. Literature will be limited to published full-text articles in the English language. There will be no restriction on settings in which the study was conducted, intervention, comparator, or the type of outcome.

### Data extraction

We will adopt part of the data extraction form from the 2007 and 2012 studies [11], with some additions to gain the information on *P* values and Bayesian analysis. We will pilot test the form using three studies from 2007 and 2017 for completeness and accuracy. Data will be extracted by a single reviewer using Excel (v. 2016, Microsoft Corporation, Redmond, WA, USA), with verification by a second reviewer. Disagreements will be resolved by discussion between reviewers or by involving another reviewer when necessary. We will extract data on characteristics of the publication, study design, intervention, control, trial conduct, study sample, sample size, hypothesis, primary objective, diagnostic criteria, recruitment strategies, funding, data monitoring committee (DMC), and specific statistical attributes of frequentist and Bayesian analysis/methods that are related to the primary outcome (see Additional file 3). We will extract data for the primary outcome, and if not clearly stated, we will use the objective outcome (e.g., mortality, hospitalization), the outcome used to calculate sample size, or the first outcome reported in the results. We will also use trial registers and published protocols (when cited in the publication) to supplement data extraction. When not cited in the publications, we will search for trial registers in the International Clinical Trials Registry Platform and the Google databases. We will not appraise the risk of bias of the included studies.

### Data analysis

We will present summary characteristics and results of all trials in a tabular form. We will consider analyzing the data using Stata (v. 16.1; StataCorp, College Station, Texas, United States) or R [16] and JAGS statistical software [17]. The analysis of extracted data will be mainly descriptive, using counts and percentages for categorical data, and means and/or medians (with standard deviations and/or ranges) for continuous data. We will compare extracted data from the 2017 sample with 300 RCTs published in 2007 to assess 10-year change in the reporting of *P* value and Bayesian analysis. The difference between the two periods will be assessed using both the frequentist and Bayesian methods. We will present the proportion (%) of studies reporting *P* value and Bayesian analysis in 2007 and 2017 in graphical forms. We will also present specific characteristics of studies, which used Bayesian analysis in tabular forms, if any. We will present the clustering around *P* values of significance, if observed in the samples.

## Discussion

To the best of our knowledge, this will be the first review to investigate the change in *P* value and Bayesian analysis in RCTs in child health research. This review will provide data on the methodological quality of RCTs in child health research, especially in the magnitude and direction of change in *P* value and Bayesian analysis in the 600 RCTs to be included in this review. Our experience with the two previous reviews will provide adequate guidance for study selection, data extraction, and interpretation of the results. We anticipate a considerable variation in the use of NHST and Bayesian methods in the 300 RCTs. Although the search strategy was clearly defined, we anticipate some limitations due to our inclusion criteria. Relevant studies may be omitted if not indexed in the databases we searched, full-text not available, or if reported in other languages other than English.

In conclusion, this review will provide robust evidence on the state of inferential statistics in RCTs in child health research. It has the potential to help inform which methodological approach should be adopted between NHST and Bayesian methods in RCTs in child health research.

### Study dissemination

We will submit reports from this study for peer-reviewed publication in appropriate academic journals. Our findings will be presented at provincial, national, and international scientific conferences and webinars. We will also share our findings via our institutional Twitter accounts.

## Supplementary Information

**Additional file 1.** PRISMA – P 2015 Checklist

**Additional file 2.** Search Strategy

**Additional file 3.** Data Extraction Guidelines

## Data Availability

Not applicable

## Abbreviations

NHST: Null hypothesis significance test
RCT: Randomized-controlled trials
