# Supplementary material for: P value and Bayesian analysis in randomized-controlled trials in child health research published over 10 years, 2007 to 2017: a methodological review protocol
Source: Syst Rev. 2021 Mar 10;10:71. doi: 10.1186/s13643-021-01622-8 (PMC7948362; doi:10.1186/s13643-021-01622-8)
Supplement: Supplementary file 2 — Additional file 2. Search Strategy [file 13643_2021_1622_MOESM2_ESM.pdf]

**Appendix A.** Search strategy

<u>Database:</u> Cochrane Central Register of Controlled Trials (Wiley)

#1- (Infant* or infancy or Newborn* or Baby* or Babies or Neonat* or Preterm* or Prematur* or Postmatur* or Child* or Schoolchild* or School age* or Preschool* or Kid or kids or Toddler* or Teen* or Boy* or Girl* or Minors* or Pubert* or Pubescen* or Prepubescen* or Pediatric* or Paediatric* or Peadiatric* or Nursery school* or Kindergar* or Primary school* or Secondary school* or Elementary school* or High school* or Highschool*):ti,ab,kw

#2- (Adolesc*):ti,ab

#3- (Infant or Child or Minors or Puberty or Pediatrics or Schools):kw

#4- #1 or #2 or #3

#5 adolescent*:kw

#6- (adolescent* and (adult* or elderly or "middle aged" or "aged, 80 and

over")):kw

#7- #6 and not #4

#8- #4 or #5

#9- #8 and not #7 from 2017 to 2017, in Trials

******************************************************************************
