# Supplementary material for: P value and Bayesian analysis in randomized-controlled trials in child health research published over 10 years, 2007 to 2017: a methodological review protocol
Source: Syst Rev. 2021 Mar 10;10:71. doi: 10.1186/s13643-021-01622-8 (PMC7948362; doi:10.1186/s13643-021-01622-8)
Supplement: Supplementary file 3 — Additional file 3. Data Extraction Guidelines [file 13643_2021_1622_MOESM3_ESM.pdf]

Bayesian/Frequentist Data Extraction Guidelines

Data Extraction Guidelines:

***Use ALL AVAILABLE INFORMATION (including protocols, companion articles references in the publication, and records of trial registration) to complete data extraction. We will include a MAXIMUM OF THREE sources per trial:**

1. The trial identified as part of our sample;
2. The trial register, if available; and
3. EITHER the published protocol or methods document, if cited in our original study (first choice) OR the sentinel trial in the case of multiple publications, if cited in our original study (second choice).

If trial registration is not declared, search, in order, the trial title/key words, corresponding author, first author, and/or last author in each of:

1. ICTRP (apps.who.int/trialsearch/)
2. Current Controlled Trials (www.controlled-trials.com/mrct/ -- select all 5 registers included in the meta-register)
3. Google

**Field**	**Responses**	**Comments**
Study ID	Free text	
First author, year of publication	Free text	
Study period (month and year)	Free text	
Study type	▪ Efficacy/superiority<br>▪ Equivalence<br>▪ Non-inferiority<br>▪ Other (specify)<br>▪ Unclear	**Efficacy/Superiority**: A study in which the authors intended to demonstrate a significant difference between treatments.<br><br>**Equivalence**: A study in which the authors intended to show that there was no significant difference between treatments.<br><br>**Non-inferiority**: A study in which the authors intended to show that the new treatment effect is not worse than the standard treatment effect.
If other, specify:	Free text	Specify study type if selected Other (specify) above.
RCT type	▪ Parallel<br>▪ Crossover<br>▪ Factorial<br>▪ Split body<br>▪ Cluster<br>▪ Other (specify)	**Parallel:** A trial that compares two groups of people concurrently, one of which receives the intervention of interest and one of which is a control group. Some parallel trials have more than two comparison groups and some compare different interventions without including a non-intervention control group (also called independent group design).<br><br>**Crossover:** A type of clinical trial comparing two or more interventions in which the participants, upon completion of the course of one treatment, are switched to another. For example, for a comparison of treatments A and B, the participants are randomly allocated to receive them in either the order A, B or the order B, A. Particularly appropriate for study of treatment options for relatively stable health problems. The time during which the first intervention is taken is known as the first period, with the second intervention being taken during the second period.<br><br>**Factorial:** A trial design used to assess the individual contribution of treatments given in combination, as well as any interactive effect they may have. Most trials only consider a single factor, where an intervention is compared with one or more alternatives, or a placebo. In a trial

Field	Responses	Comments
		using a 2x2 factorial design, participants are allocated to one of four possible combinations. For example in a 2x2 factorial RCT of nicotine replacement and counselling, participants would be allocated to: nicotine replacement alone, counselling alone, both, or neither. In this way it is possible to test the independent effect of each intervention on smoking cessation and the combined effect of (interaction between) the two interventions. This type of study is usually carried out in circumstances where no interaction is likely.<br><br>**Split body:** A trial in which separate body parts within each participant (e.g., eyes, teeth) were randomized to receive or not receive an intervention.<br><br>**Cluster:** A trial in which pre-existing groups of participants (e.g., schools, villages) are randomly selected to receive or not receive an intervention.
If other, specify:	Free text	Specify RCT type if selected Other (specify) above.
Country	Free text	If country not reported then NR.
Study hypothesis reported	▪ Yes<br>▪ No<br>▪ Unclear	May or may not be explicit. Does not necessarily have to contain the word hypothesis, but a clear statement of the investigators' stance before commencing the study.
Type of study hypothesis	▪ Null hypothesis (frequentist)<br>▪ Null hypothesis + a priori (frequentist)<br>▪ A priori or alternative hypothesis or additional evidence (Bayesian)<br>▪ Other (specify)<br>▪ N/A	**Null hypothesis:** Hypothesis that there will be no significant difference between the groups; can be a statement of equality, of no relationship, or of no causal relationship.<br><br>**Null hypothesis + a priori:** Null hypothesis + consideration for previous evidence or distribution or probability.<br><br>**Alternative hypothesis:** A second hypothesis is given that is different from the null hypothesis; does not necessarily have to be the exact opposite of the null (e.g. there is a difference between the groups); for example, the variable may have an effect at high dosages but not low<br><br>**Additional evidence:** Prior evidence from literature or experts was used/considered when determining a priori hypothesis
Other (specify)	Free text	Specify hypothesis type if selected Other (specify) above, e.g. studies that report on one treatment method being better than the other (superiority studies).
Primary objective of the study	Free text	As reported by the study authors
Primary diagnostic category	▪ Infectious and parasitic diseases<br>▪ Neoplasms<br>▪ Diseases of the blood or blood-forming organs<br>▪ Diseases of the immune system<br>▪ Endocrine, nutritional, or metabolic diseases<br>▪ Mental, behavioural, or neurodevelopmental disorders<br>▪ Sleep-wake disorders<br>▪ Diseases of the nervous system	Select the primary diagnostic category of the study using the ICD-11 classification system: https://icd.who.int/browse11/l-m/en

Field	Responses	Comments
	▪ Diseases of the visual system<br>▪ Diseases of the ear or mastoid process<br>▪ Diseases of the circulatory system<br>▪ Diseases of the respiratory system<br>▪ Diseases of the digestive system<br>▪ Diseases of the skin<br>▪ Diseases of the musculoskeletal system or connective tissue<br>▪ Diseases of the genitourinary system<br>▪ Conditions related to sexual health<br>▪ Pregnancy, childbirth, or the puerperium<br>▪ Conditions originating in the perinatal period<br>▪ Symptoms, signs, or clinical findings<br>▪ Injury, poisoning, or consequences of external causes<br>▪ External causes of morbidity or mortality<br>▪ Factors influencing health status or contact with health services<br>▪ N/A	
Intervention class	▪ Drug<br>▪ Vaccine<br>▪ Rehabilitation/psychosocial<br>▪ Prevention/screening<br>▪ Surgery/radiotherapy<br>▪ Communication, organizational, or educational<br>▪ Alternative therapeutic<br>▪ Device<br>▪ Other (specify)	
If other, specify:	Free text	Specify intervention class if selected Other (specify) above.
Control type	▪ No intervention<br>▪ Placebo<br>▪ Active intervention<br>▪ Wait-list control<br>▪ Other (specify)	Select all that apply
If other, specify:	Free text	Specify control type if selected Other (specify) above.
Number of centres	▪ Single centre	

Field	Responses	Comments
	▪ Multicentre<br>▪ Unclear	
Recruitment strategies used	▪ Standard<br>▪ Non-standard	**Standard:** Participants provide consent and then are randomized in a typical manner (i.e., 1:1, 2:1, etc.).<br><br>**Not standard:** Any of:<br>▪ Open trial design (randomization occurs before participants are approached, so that participants are informed of the treatment they were randomized to receive prior to consent)<br>▪ Increasing or decreasing the chance of receiving the experimental treatment<br>▪ Experimental treatment for all participants and standard treatment for non-participants<br>▪ Standard care for all participants and experimental treatment for non-participants<br>▪ Random assignment of treatment for participants and choice of treatment for non-participants<br>▪ Opt in or opt out recruitment (consent was sought for participation or non-participation)
Number of randomized arms	Free text	
Number of sample size enrolled	Free text	Use NR if applicable
Number randomized	Free text	Use NR if applicable
Number analyzed	Free text	Use NR if applicable
Power of the trial calculated	▪ Yes<br>▪ No	Answer “yes” if the authors report the **actual power of the study**– distinct from using the desired power to calculate the sample size.
Sample size calculated	▪ Yes<br>▪ No	In relation to the primary objective of the study.
Source of funding reported	▪ Yes<br>▪ No	
Funding source type	▪ Government<br>▪ Academic or research institute<br>▪ Private<br>▪ Pharmaceutical<br>▪ Industry for device<br>▪ No external funding<br>▪ Unclear<br>▪ Other (specify)<br>▪ N/A	Select all that apply.<br><br>Canadian Institutes of Health Research (CIHR) and National Institutes of Health (NIH) are considered government funding.<br><br>If a foundation is listed as a funding source, report as “Private”.<br><br>Include all United Nations agencies under “Other”.
If other, specify:	Free text	Specify funding source type if selected Other (specify) above.
Data monitoring and safety reported	▪ Yes<br>▪ No	May be referred to as a Data Monitoring Committee (DMC), Data Monitoring Safety Board (DSMB), or Data Monitoring Board (DMB).
Trial continuity/termination based on	▪ Bayesian, credible interval<br>▪ P-value only (frequentist)<br>▪ 95% confidence interval<br>▪ Other (specify)<br>▪ N/A	**Bayesian, credible interval:** When the decision to continue or terminate the study is supported by a prior evidence/distribution or literature, evidence from experts, or when similar studies were terminated, or via interval generated from prior distribution/probability<br><br>**Credible interval:** Bayesian equivalent of the confidence interval that is based on a priori evidence; probability that the given parameter is within the specified range

Field	Responses	Comments
		**P-value only:** Deciding to continue or terminate the study is based only on observed results in the given study and P-value calculations<br><br>**95% confidence interval:** Deciding to continue or terminate the study is based only on observed results in the given study and the calculated 95% confidence interval
If other, specify:	Free text	Specify trial continuity/termination based on if selected Other (specify) above.
Primary outcome	Free text	Specify the primary outcome.<br><br>When the primary outcome is not stated explicitly by the study authors:<br><br>1. Choose the objective over the subjective outcome.<br>2. If the sample size calculation is based on an outcome, use it as the primary outcome.<br>3. If 1 and 2 aren't met, use the first outcome listed in the Results section as the primary outcome.
Primary outcome category	▪ Behavioural<br>▪ Biomarker<br>▪ Pain<br>▪ Physiological<br>▪ Psychological<br>▪ Techniques/training<br>▪ Quality of life<br>▪ Other (specify)	Based on the primary outcome as reported by the authors or inferred by the reviewer.<br><br>**Behavioural:** e.g. attitudes, eating behaviours<br>**Biomarker:** NIH definition: A characteristic that is objectively measured and evaluated as an indicator of normal biologic processes, pathogenic processes, or pharmacologic responses to a therapeutic intervention. E.g. blood glucose, urine cultures.<br>**Pain:** e.g. pain relief, pain prevention<br>**Physiological:** adapted from NIH definition: A characteristic or variable that reflects how a patient feels, functions, or survives. E.g. disease progression, mortality<br>**Psychological:** e.g. depression assessment scores, neuropsychological test performance<br>**Techniques/training:** method of intubation, effectiveness of a focus group<br>**Quality of life:** e.g. SF-36, patient satisfaction
If other, specify:	Free text	Specify primary outcome category if selected Other (specify) above.
Report on 95% confidence interval for primary outcome	▪ Yes<br>▪ No	
Report on effect size for primary outcome	▪ Yes<br>▪ No	Studies that report the quantitative measure of the degree of an effect/change on the primary outcome of interest, such as the strength of odds ratio, relative risk, or proportion.
Primary outcome analysis based on	▪ Any Bayesian inferential statistics<br>▪ P-value only (frequentist)<br>▪ 95% confidence interval<br>▪ P-value + any Bayesian inferential statistics<br>▪ Other (specify)	**Any Bayesian inferential statistics:**<br>**A priori probability:** The probability of the outcome from previous findings or evidence<br><br>**A priori distribution:** The distribution of the outcome from previous findings or other evidence<br><br>**A priori effect estimate:** E.g. HR, RR, OR, mean difference, regression coefficient, etc.; when the effect estimate from previous findings are considered<br><br>**Posterior probability:** Combination of a prior probability and the probability of observed data in a given study

Field	Responses	Comments
		**Posterior distribution:** Combination of a prior distribution and that of the observed data in a given study<br><br>**Posterior effect estimate:** E.g. HR, RR, OR, mean difference, regression coefficient, etc.; the effect estimate that is the combination of the a priori effect estimate and the observed data from the given study<br><br>**Credible interval** – see above<br><br>**95% confidence interval:** Primary outcome analysis is based only on a 95% confidence interval that was calculated based only on the observed data from the given study
If other, specify:	Free text	Specify primary outcome analysis based on if selected Other (specify) above.
Effect estimate for the primary outcome	▪ Median ±IQR<br>▪ Mean ±SD<br>▪ Mean difference<br>▪ Odds ratio (OR)<br>▪ Hazard ratio (HR)<br>▪ Risk ratio (RR)<br>▪ Probability<br>▪ Percentage<br>▪ Other (specify)<br>▪ N/A	Qualitative measure of the degree of an effect/change on the primary outcome of interest, e.g. mean difference between two groups, odds ratio of developing outcome, percentage/proportion of patients that improved.
If other, specify:	Free text	Specify effect estimate for the primary outcome if selected Other (specify) above.
Interim analysis reported	▪ Yes<br>▪ No	Analysis that is conducted before the completion of data collection.
Sensitivity analysis based on	▪ Any Bayesian inferential statistic<br>▪ P-value only (frequentist)<br>▪ 95% confidence interval<br>▪ P-value + any Bayesian inferential statistic<br>▪ Other (specify)<br>▪ N/A	Analyzing if the results would change if some studies or participants were excluded, if missing data was treated differently, or if a more conservative estimate of the treatment effect was used.<br>See above
If other, specify:	Free text	Specify sensitivity analysis based on if selected Other (specify) above.
Primary outcome interpretation based on 95% confidence interval	▪ Yes<br>▪ No	Interpretation will be in the discussion section.
Primary outcome interpretation based on effect size	▪ Yes<br>▪ No	See above
Primary outcome interpretation based on	▪ Bayesian inferential statistics<br>▪ P-value only (frequentist)<br>▪ P-value + any Bayesian inferential statistic<br>▪ Other (specify)	See above

Bayesian/Frequentist Data Extraction Guidelines

Field	Responses	Comments
If other, specify:	Free text	Specify primary outcome interpretation based on if selected Other (specify) above.
P-value level of significance for primary outcome	Free text	As reported by the study authors.
Results interpretation for primary outcome based on observed data only	▪ Yes<br>▪ No	"No" if interpretation is based on observed data + introduction of a priori data (e.g. a priori probability, prior HR, prior OR, prior coefficient) or previous scientific evidence (e.g. scientific conference data, expert testimony, etc.) that will alter the interpretation of the results. This is commonly found in the methods or results sections. In the results section, look for any of the Bayesian inferential statistics (e.g. a priori probability, prior HR, prior coefficient).
Bayesian inferential statistics reported	▪ Yes<br>▪ No	
Types of Bayesian inferential statistics	▪ A priori probability<br>▪ A priori distribution<br>▪ A priori effect estimate<br>▪ Posterior probability<br>▪ Posterior distribution<br>▪ Posterior effect estimate<br>▪ 95% credible interval<br>▪ Other (specify)<br>▪ N/A	See above. Select all that apply.
If other, specify:	Free text	Specify types of Bayesian inferential statistics if selected Other (specify) above.
Conclusion	▪ Bayesian<br>▪ Frequentist<br>▪ Bayesian + frequentist	Reviewer's conclusion regarding category of the study.<br>**Bayesian:** Bayesian inferential statistics consider a priori evidence in addition to the observed data when making conclusions. Use of any Bayesian inferential statistic or methods in methods, analysis, and/or results.<br><br>**Frequentist:** Use of p-value in methods, analysis, and/or results.<br><br>**Bayesian + frequentist:** Use of at least one Bayesian inferential statistics in the methods (including hypothesis testing), analysis, or results section.
MCID reported	▪ Yes<br>▪ No	**MCID** – minimal clinically important difference
Notes	Free text	Any information the reviewer would like to highlight.
